# Brain mechanisms underlying the modulation of heart rate variability when accepting and reappraising emotions

**DOI:** 10.1038/s41598-024-68352-4

**Published:** 2024-08-13

**Authors:** Simón Guendelman, Laura Kaltwasser, Mareike Bayer, Vittorio Gallese, Isabel Dziobek

**Affiliations:** 1 Clinical Psychology of Social Interaction, Institute of Psychology, Humboldt-Universität Zu Berlin, Berlin, Germany; 2 Berlin School of Mind and Brain, Humboldt-Universität Zu Berlin, Berlin, Germany; 3 Department of Medicine & Surgery, Unit of Neuroscience, University of Parma, Parma, Italy; 4 Italian Academy for Advanced Studies in America, Columbia University, New York, USA

**Keywords:** Heart rate variability, Emotion regulation, Cognitive reappraisal, Acceptance, fMRI, Neuro-imaging, Psycho-physiology, Psychology, Human behaviour, Emotion, Autonomic nervous system

## Abstract

Heart rate variability (HRV) has been linked to resilience and emotion regulation (ER). How HRV and brain processing interact during ER, however, has remained elusive. Sixty-two subjects completed the acquisition of resting HRV and task HRV while performing an ER functional Magnetic Resonance Imaging (fMRI) paradigm, which included the differential strategies of ER reappraisal and acceptance in the context of viewing aversive pictures. We found high correlations of resting and task HRV across all emotion regulation strategies. Furthermore, individuals with high levels of resting, but not task, HRV showed numerically lower distress during ER with acceptance. Whole-brain fMRI parametrical modulation analyses revealed that higher task HRV covaried with dorso-medial prefrontal activation for reappraisal, and dorso-medial prefrontal, anterior cingulate and temporo-parietal junction activation for acceptance. Subjects with high resting HRV, compared to subjects with low resting HRV, showed higher activation in the pre-supplementary motor area during ER using a region of interest approach. This study demonstrates that while resting and task HRV exhibit a positive correlation, resting HRV seems to be a better predictor of ER capacity. Resting and task HRV were associated with ER brain activation in mid-line frontal cortex (i.e. DMPFC).

## Introduction

### Emotion regulation: reappraisal and acceptance

A key factor associated with resilience and mental health is emotion regulation (ER), the capacity to modify emotional state which has been described as one sub-component of emotional intelligence^1–3^. During stressful circumstances ER abilities facilitate the down-regulation of distress levels at the psychological (i.e. self-reported) and physiological levels. Literature has distinguished between maladaptive ER strategies, as being linked to psychopathology, and adaptive ER strategies, as precursors of mental health^4^. The most studied adaptive ER strategies include reappraisal (modifying the cognitive frame of the stressful event) and mindful acceptance (non-reactively allowing the emotional state)^5,6^.

From a psychological point of view, both strategies engage different cognitive resources (cognitively reframing emotional stimuli vs accepting it as is), and thus they might be realized via different implementation of brain and physiological processes. In fact, the only study that directly compared these ER strategies showed that reappraisal was more effective in reducing distress but was more resource-consuming in terms of cognitive, brain (higher activation in regulatory regions, such as the prefrontal cortices), and physiological processing (higher heart rate), suggesting higher metabolic costs^7^. Given both are central part of current therapeutic approaches (reappraisal in cognitive behavioral therapy—CBT, and acceptance in acceptance and commitment therapy—ACT) the further understanding of their neurophysiological basis is crucial to advancing the comprehension of action mechanisms of psychological interventions^8^.

### Heart rate variability and socio-emotional functioning

Heart rate variability (HRV), the degree of variation in heart beating, has been linked to a myriad of mental health outcomes (i.e., resilience). Previous literature has shown that higher levels of HRV are linked to higher executive^9^, cognitive^10^ and social functioning^11,12^, and also lower levels of HRV have been shown in diverse medical and mental health conditions such as anxiety or mood disorders^13–15^. HRV (here we will use ‘HRV’ to refer to what usually is the High-Frequency range of HRV), has been proposed as one physiological marker for the ‘parasympathetic system’ activity—a branch of the autonomic nervous system -, which together with the sympathetic system exerts control over diverse visceral functions, including heart rate, respiratory frequency, inflammation, among others^16^. The high frequency domain of HRV, in the context of frequency domain analysis, has been suggested to offer the best representation of the parasympathetic tone, as it has been shown in previous studies^17,18^. Different accounts have posited a central role to the parasympathetic system, i.e. the neurovisceral integration theory by Thayer and Lane (2000), and the polyvagal theory by Stefen Porges^20^, specially connecting high-order and volitional self-regulation processes and the adaptive heart rate regulation, via brain (middle) frontal regions (Thayer, Åhs, Fredrikson, Sollers, & Wager^21^). This role of linking brain cortical with autonomic heart modulation might be critical for ER processing, which—by definition—includes the modification of both psychological and physiological levels of stress.

### Heart rate variability as a marker of emotion regulation

Several studies have thus specifically linked HRV and ER. Using resting HRV, acquiring HRV measurements during a rest condition, studies using behavioral and self-reported instruments have shown that subjects with higher resting HRV have lower daily stress^22^, and better ER of negative emotions^23^, overall depicting more adaptive ER and flexible responding strategies^24^. On the other hand, studies investigating ER and task HRV, acquiring HRV measurements during a task condition, have shown a similar pattern, suggesting that the higher task HRV is linked to higher self-regulation efforts or stress recovery^24^. Nevertheless, to the best of our knowledge, no previous study has investigated both resting and task HRV during ER, while also taking into consideration their brain mechanisms.

### Brain processes underlying heart rate variability and emotion regulation

Studies investigating the neurobiological basis of ER, using tasks in which subjects have to explicitly downregulate negative emotions, have shown a privileged engagement of areas like the dorsal and ventral parts of the lateral prefrontal cortex (DLPFC, VLPFC), but also of mid-line regions of the prefrontal cortex (PFC), like the pre-supplementary motor area (pre-SMA), anterior cingulate cortices (ACC), among others^25–27^. Similarly, studies examining the relationship between HRV, albeit mixing resting and task HRV, and brain activation during cognitive and emotion control tasks, have shown a positive relationship between HRV and the insula and mid-line regions of PFC (i.e. medial PFC, pre-SMA and ACC; meta-analyses by Mulcahy, Larsson, Garfinkel, & Critchley, 2019; Thayer et al., 2012).

Moreover, few studies have examined HRV and specific ER brain processes together: a study comparing low vs high resting HRV subjects showed that the latter group had higher activation in the dorso-medial PFC (DMPFC)^29^. Another study showed that subjects with higher resting HRV had stronger connectivity between DMPFC and amygdala^30^. These studies suggest that resting HRV is associated with brain mechanisms underlying emotion regulation. Nevertheless, these studies have several methodological shortcomings, including small sample size, not assessing both task and resting HRV, reporting of uncorrected *p*-values, etc. (i.e. Steinfurth et al., 2018). Interestingly, so far, no studies have examined the relationship between resting and task HRV and ER from a behavioral and brain perspective of embodied interaction. More specifically, how the brain and heart interact during ER, has remained elusive.

### Brain processes underlying the modulation of heart rate variability during emotion regulation

Hence, in the current study we sought to further elucidate the relationship between resting and task HRV and ER at the behavior and brain level, and more specifically set out to test the hypothesis that subjects with higher resting HRV also show higher task HRV during an ER task. Likewise, we sought to investigate whether levels of resting and task HRV moderate emotion regulation capacities, hypothesizing that subjects with higher resting and task HRV will display lower personal distress when regulating own emotions more generally, thus, when using both reappraisal and acceptance strategies.

At the brain level, and using task HRV as a parametric regressor in first-level analyses, we predicted increased brain—HRV covariation during ER with reappraisal in mid-line brain regions, like the dorso-medial PFC, ventral ACC and pre-SMA (Thayer et al., 2012). Using the same approach, we predicted increased brain—HRV covariation during ER with acceptance in mid-line brain regions, like the dorso-medial PFC, ventral ACC and pre-SMA, but also in insular and parietal cortices, linked to somato-sensory processing (Goldin et al., 2019; Thayer et al., 2012). Thus, brain—task HRV covariation will be especially evident in ER brain regions for both strategies.

In addition, using resting HRV as a between-subject covariate for higher-level analyses, we predicted that subjects with higher resting HRV would show higher brain activation in the same mid-line regions, especially in those involved in both ER and HRV modulation (i.e. pre-SMA), when contrasting both reappraisal and acceptance to a baseline condition^29^. Thus, we attempt to replicate previous findings associating resting HRV and ER brain activation.

Finally, to further investigate differences between reappraisal and acceptance, we took an exploratory approach and contrasted task HRV brain activation covariation maps between both strategies. Here we expected that reappraisal over acceptance would lead to higher brain—HRV covariation in mid-line regions (DMPFC), whereas contrasting acceptance with reappraisal would lead to higher brain—HRV covariation in somato-sensory regions (i.e., inferior parietal lobule, temporo-parietal junction), as acceptance entails volitional engagement with current bodily experience^5,6^. Thus, these analyses would further the understanding of the differential neurophysiological substrates between both strategies.

To evaluate these questions, we took advantage of a dataset previously acquired in the context of a mindfulness intervention study^31^, implementing an emotion regulation task (ERT), in which subjects had to alternate between regulating their own emotions with reappraisal and acceptance and a baseline condition (permitting emotions), while being exposed to aversive pictures. Subjects completed the ERT in the magnetic resonance scanner, while self-report stress ratings and heart rate were measured.

## Methods

### Participants

Sixty-two healthy subjects (52 female, mean age = 38.5 years, sd = 10) were selected from the general population in the city of Berlin in the context of a longitudinal study on the effects of a Mindfulness based intervention, pre-registered at clinicaltrials.gov NCT03035669. Only data that were collected before randomization into the longitudinal trial will be reported here. The longitudinal data was reported on elsewhere^31^. Subjects completed an online and in-person screening procedure, inclusion criteria were as follow: no history of neurological or psychiatric disorders, no current use of psychoactive drugs, native German fluency, normal or corrected-to-normal vision and no contra-indication for performing an fMRI experiment. All participants provided written informed consent prior to the investigation. The study was approved by the ethics committee of Humboldt-Universität zu Berlin and was carried out in accordance with the guidelines of the declaration of Helsinski.

### Procedure

Participants underwent an emotion regulation paradigm in the context of an fMRI experiment on day one and completed various socio-emotional measures on day two. The experiment took place between 9:00 and 19:00 h. during the day.

### Emotion regulation task (ERT)

In this emotion regulation task (ERT), participants were required to complete separate blocks for regulating own (ER) and regulating others’ emotions, while being exposed to aversive pictures, the experimental task has been described in previous publications^31,32^. According to the scope and hypotheses, for this study, only blocks pertaining self ER are reported, while blocks for regulating others’ emotions are reported on in a separate publication^32^. The strategies subjects were asked to engage in included reappraisal and acceptance as emotion regulation methods, and permitting of any emotional state as a baseline condition. Before entering the scanner, subjects were carefully trained by the experimenters in each specific strategy, reappraisal was introduced as an “emotional distancing from stressful stimuli”, acceptance as “bringing a sense of gentleness and kindness to the present experience”, and permit as “not trying to decrease the negative emotional state”. Participants could only enter the scanner after successfully understanding and implementing each strategy. Throughout the task the three strategies were applied for the ER blocks, resulting in three different conditions. To prevent systematic spill-over effects, all blocks were presented in a random and counterbalanced order. In addition, inter block intervals provided enough time for stimulus BOLD signal response to decay. The strategies were applied via verbalization of specific sentences that were displayed on the photos for every condition (as for the regulation conditions with reappraisal: “This is just a photo!”, with acceptance: “gently accept!”; as for the non-regulation conditions with permit: “Permit the reaction!”). Subjects were told to not only read the sentences, but to really engage and actively apply the respective strategies to down-regulate (or permit) their own current negative emotional state. Subjects had to verbalize the strategy during the first two seconds of the picture, in order to standardize implementation across subjects and conditions. Subjects self-instructed aloud the strategy (reappraisal: “I see, it’s just a photo”; acceptance: “I gently accept”) as means for reducing own distress. During the permit condition subjects were asked to also verbalize to themselves to permit the respective emotions (permit: “I permit the reaction”). They were instructed to not try to either regulate (decrease) or to worsen distress (increase). Since this is a non-regulation condition, we expect higher distress levels. In addition, conditions were kept as similar as possible to control for visual processing when contrasting them.

**Figure 1:**
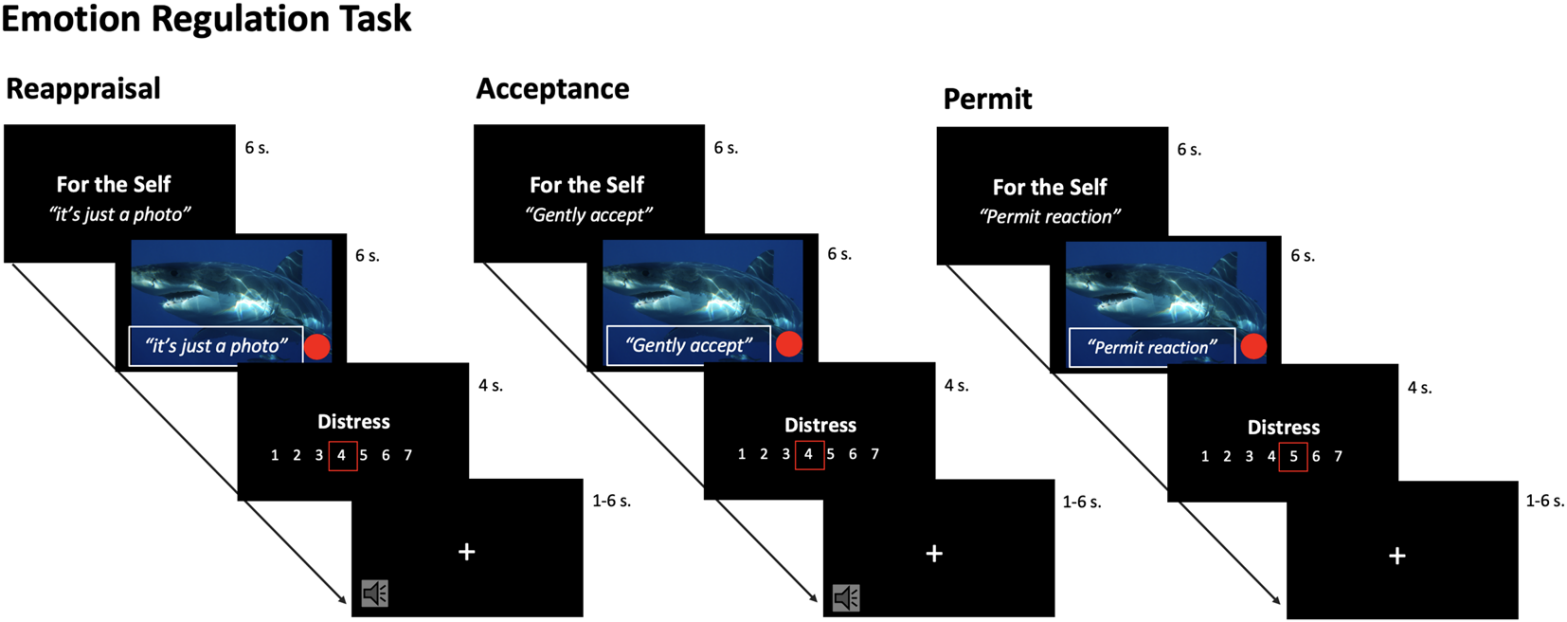
Schematic representation of the ERT experimental set-up in the fMRI scanner. In the experiment, the subject in the scanner (the regulator), alternates between two strategies (reappraisal vs. acceptance) and permit, resulting in three conditions: *emotion regulation* via *reappraisal* (“I see, it’s just a photo”), *emotion regulation* via *acceptance* (“I gently accept”), *emotion permitting* (“I permit the reaction”).

Subjects were informed that their physiological arousal state was constantly monitored during the experiment and that if they failed to regulate themselves, there would be the possibility of an aversive sound appearing at the end of the block, which was intended to motivate volitional effort and decreasing habituation throughout the task. A pumping red dot, which was displayed on the photographs (see Fig. 1), allegedly represented the arousal state of the subject in the scanner during the ER trials.

The pumping dot frequency was calculated a priori, according to the normative arousal values of the respective International Affective Picture System (IAPS) stimuli^33^, so it was equal across conditions for all subjects. The pumping dot aimed to increase the salience of the arousal state and thus the motivation of performing ER. Subjects were explicitly told that no aversive sound would occur during the permit condition and that self-reported stress ratings had no effect on the likelihood of getting the aversive sound throughout the whole experiment. The aversive sound was customized for every subject at the beginning of the scanner session, never overpassing the individual’s accepted intensity threshold. For standardization purposes every subject was randomly assigned to receive a fixed number of 3 to 5 aversive sounds during the whole experiment (See Table S1). Overall, the aversive sound was counterbalanced across subjects (for more information about the paradigm, see previous publications^31,32^.

The aversive pictures used in the ERT were selected from the International Affective Picture System (IAPS)^33^, including neutral and negatively valenced pictures of high and low arousal. Selection was based on normative valence and arousal mean values (for a list of pictures and mean values for arousal and valence, see Table S2 in the supplementary materials).

Stimuli were presented in a blocked event-related design with 4 blocks for each condition (*emotion regulation*-*reappraisal*, *emotion regulation*-*acceptance, emotion permitting*). Every block started with a 6 s introductory screen indicating the block type e.g. “For the self: I see it’s just a photo”, corresponded to regulate own distress with reappraisal, followed by the interleaved presentation of 6 pictures (2 neutral, 2 negative high arousal, 2 negative low arousal) for 6 s each. Participants had 4 s to rate their own distress level after every picture. At the end of each block a fixation cross was presented, during this period the aversive sound could appear (See Fig. 1). The fixation cross was jittered (mean 2000 ms, SD 1635 ms; with a MatLab script) using an exponential distribution of random numbers, accounting for the repetition time and stimulus presentation, optimizing power and efficiency for sampling the hemodynamic response in our experimental design (cf.^34^).

### Data acquisition and preprocessing

#### Heart rate variability

During the experiment, heart rate was collected with a biopac MP-150 system with a sampling rate of 100 Hz. A photo-pletismography pulse detector device was placed on the left middle finger. Heart rate peaks were detected using the automatic multiscale-based peak algorithm^35^. Inter peak distances were analyzed with NeuroKit.py, version 0.1.99^36^ to compute the spectral power in the frequency band for high frequency heart rate variability, for resting HRV acquisition and task HRV acquisition (for every trial along the ER experimental conditions). NeuroKyt implements a Hilbert transformation for estimating the amplitude of each frequency domain separately over time, the (high) frequency domain from 0.15 to 0.40 Hz was selected which indexes parasympathetic tone. In case the inter-peak distance in an area was lower than 80 ms a combinatorial optimization approach was used to select the peak that maximizes the distance to all other peaks (i.e. the peak closest to the center). While inter-peak distance higher than 4 SD (SD corresponded to the empirical standard deviation of the inter-peak distances) resulted in the exclusion of those values. Despite photoplethysmography-derived indices being less established compared to standard electrocardiography (ECG) acquisition, basic studies have found very high correlations between photoplethysmography and ECG-derived HRV metrics, including standard RMSSD and high frequency HRV^18,37^.

Resting HRV was estimated from the HR acquisition during the resting state fMRI task that occurred immediately after the ERT and lasted 7 min. Task HRV was estimated from the HR acquisition during the ER fMRI task, during the 6 s subjects regulated their emotions.

#### fMRI

Brain images were collected with a Siemens 3 Tesla Trio scanner, using a 32-channel head coil. Acquisition of structural brain images used a Magnetization-prepared rapid acquisition gradient echo (MPRAGE) sequence (1 × 1 × 1 mm^3^ resolution), with TE = 2.52 ms, TR = 1900 ms, flip angle = 90° and 100% field of view. During the ER task, whole brain T2*-weighted functional images were acquired with a standard EPI (Echo-Planar Imaging) sequence for fMRI, with TE = 30 ms, TR = 2000 ms, flip angle = 90°, field of view 100%, with an acquisition matrix = 64 × 64 × 33 slices, slice thickness of 3.75 mm, width and depth of 3 mm, with descending slice acquisition; a total of 1050 volumes were acquired per subject.

Pre-processing and analysis of data was done using FSL 6.0 (FMRIB's Software Library, www.fmrib.ox.ac.uk/fsl). Head motion correction was performed using rigid-body transformations based on a linear registration tool^38^. Images were spatially smoothed with a Gaussian kernel of 5 mm (FWHM) and high-pass filtered with a cutoff of 100 s. Functional EPI images were registered to the high-resolution structural MPRAGE images using boundary-based registration (BBR) and to standard space images (MNI_152, 2 mm resolution) using affine linear transformation with 12 degrees of freedom using FLIRT^39^. This linear approach has shown to produce accurate and robust normalization to standard space^38–40^, it is still being used in the field^30,41^.

Finally, a quality control of the mean frame displacement, signal to noise ratio and standard deviation of signal over voxels was performed for each subject. Based on these, two participants were discarded due to excessive movement (mean absolute displacement > 1.5 mm).

### Data analyses

#### Behavioral data

To evaluate the effects of emotion regulation strategies on stress levels during the ERT, a one factor (Strategy: Reappraisal vs. Acceptance vs. Permit) repeated measures analysis of variance (ANOVA) was performed. In posthoc comparisons, *p*-values were corrected using family wise error correction (FWE), with Holm-Bonferroni procedure. Parts of this data has been reported already in a previous publication and are not reported here, specifically the blocks of regulating other’s emotions^31,32^. In this publication we solely report conditions pertaining to regulating own emotions. To evaluate the effects of emotion regulation strategies on task HRV levels during the ERT, a one factor (Strategy: Reappraisal vs. Acceptance vs. Permit) repeated measures analysis of variance (ANOVA) was performed. In posthoc comparisons, *p*-values were corrected using FWE with Holm-Bonferroni procedure.

In order to elucidate the relationship between ER (stress ratings) and HRV measurements, a correlational analysis (using one-tailed *Spearman’s ρ* correlations) on self-reported stress levels, resting HRV and task HRV as variables of interesting was performed. Spearman’s ρ was chosen because it is more robust to outliers and skewed distributions, therefore accounting for the non-normal distribution of the resting HRV data (for details, see results section). Behavioral data were analyzed using IBM SPSS Statistics software, version 21.

#### fMRI

Preprocessed images were entered into a general linear model (GLM) for statistical analysis. A first level analysis at the individual level included regressors for each experimental condition (reappraisal, acceptance, permit) convolved with the canonical hemodynamic response function. The GLM modeled exclusively the BOLD signal as regressor of interesting, corresponding to the window of 6 s during which the participants were exposed to the emotional stimuli and needed to regulate or permit their emotional reactions. Complementary first level analyses additionally included parametric regressors derived from de-meaned resting and task HRV (in separate analyses), in order to investigate covariations of these parameters with the BOLD signal.

Higher-level group statistics were performed with FLAME-1, using mixed effects modeling for all first-level analyses (as fixed effects). Contrasts of interest consisted of main effects of ER strategies compared to a baseline condition of permitting emotions (ER_reap > ER_permit; ER_accept > ER_permit), as well as a direct comparison of reappraisal and acceptance (ER_reap vs ER_accept). To further compare brain -task HRV co-activation patterns of emotion regulation versus baseline (permit), covariations of brain activation and task HRV were investigated for each strategy compared to baseline (ER_reap*task_HRV > ER_permit*task_HRV, ER_accept*task_HRV > ER_permit*task_HRV) and as comparison between strategies (ER_reap*task_HRV vs ER_accept*task_HRV). Finally, the impact of resting HRV on brain activations was investigated with a group comparison (high vs. low resting HRV as between-subject factor) in contrast to baseline ((ER_reap > permit) × resting HRV); (ER_accept > permit) × resting_HRV) and compared between strategies ((ER_accept vs ER_reap) × resting_HRV). Contrasts of interest were modeled as block-designs, in order to increase the sensibility to specific brain responses across conditions (ER strategies), reduce variability (increasing signal to noise ratio), and increase statistical power. Same analytical procedure was successfully implemented in previous publications^31,32^.

Whole-brain analyses were thresholded using permutation tests (with 5000 CIs) and corrected for multiple comparisons with threshold-free cluster enhancement (TFCE) equivalent to a family-wise error correction (FWE) of < 0.05^42^.

Since analyses revealed a non-normal distribution of the resting HRV data (for details see results section), a median split criterium was used to define two groups (high and low resting HRV) for resting HRV—brain activation analyses (see suppl. material, figure S2).

Finally, to complement the whole-brain analysis approach, a region of interest (ROI) analysis was implemented as an exploratory follow-up analysis, using previously reported coordinates specific for pre-SMA region, as a potentially crucial midline brain structure for both ER and HRV modulation (ER meta-analysis, cross ref: Kohn et al., 2014), and in line with our predefined hypotheses linking ER and HRV in mid-line brain regions^25^. Starting from the centroid of the ROI (MNI Coordinates: − 2, 14, 58), we created a 5 mm diameter sphere and extracted the PE for each subject. To elucidate the effects of emotion regulation strategies and resting HRV on pre-SMA activation during the ERT, a repeated measures analysis of variance (ANOVA) with one within-subject factor (Strategy: Reappraisal vs. Acceptance vs. Permit) and one between-subjects factor (Group: high resting-HRV vs low resting-HRV) was performed. In posthoc comparisons, *p*-values were FWE—corrected.

### Statement of ethics

All research procedures, including testing in human subjects, were conducted in accordance with the World Medical Association Declaration of Helsinki. The study protocol was approved by the local ethics committee from the Institute of Psychology, Humboldt-Universität zu Berlin, Germany.

## Results

### Manipulation check

Individual interviews after the study confirmed that all subjects could complete and perform the experiment according to the instructions, they all actively applied the strategies to regulate their emotions during the experiment.

### Behavior

#### Descriptive statistics for the emotion regulation task (ERT)

Regarding the emotion regulation task, means (M) and standard deviations (SD) for self-reported distress ratings for the regulator were: for reappraisal (M = 3.17; SD = 0.87), acceptance (M = 3.36; SD = 0.78), and permitting of own emotions (M = 3.43; SD = 0.78).

#### Effects of emotion regulation strategies on self-reported distress ratings during ERT

Analyses yielded a main effect of Strategies (*F*_1,53_ = 8.26, *p* < 0.001, *η*^2^_*p*_ = 0.135), indicating a differential distress level across ER conditions. Post-hoc comparisons showed lower stress ratings for reappraisal compared to permitting emotions (corrected *p* = 0.007) and compared to acceptance (corrected *p* = 0.006), and a nonsignificant difference between acceptance and permitting (corrected *p* = 0.64) (Figure S1).

#### Effects of emotion regulation strategies on task HRV during ERT

Analyses yielded a non-significant effect of Strategies (*F*_2,120_ = 0.31, *p* = 0.74, *η*^2^_*p*_ = 0.01), indicating no differences in task HRV levels across ER conditions.

#### Descriptive statistics for resting HRV

Resting HRV described a mean of 0.034 ms^2^ (SD = 0.020), a median of 0.029, and a Shapiro–Wilk test of 0.91 (*p* < 0.05), indicating a non-normal distribution. Further inspection showed the presence of two modes (1 = 0.008; 2 = 0.05) thus suggesting a bi-modal distribution for resting HRV (Figure S2). Therefore, analyses strategies were adapted to account for this distribution by performing correlational analyses of behavioral data using Spearman’s ρ, and by using a median-split procedure for resting HRV—brain covariation analyses.

#### Association between emotion regulation distress ratings and resting and task HRV

The correlational analyses on the relationship between emotion regulation and resting HRV indicated a significant (acceptance) and near-significant (reappraisal) negative association between distress ratings (self-reported) and resting HRV for both ER strategies, but did just not remain significant after FWE adjustments (Table 1, first column). For emotion permitting and resting HRV there was a nonsignificant association. This suggests, descriptively, that subjects with higher resting HRV have significantly lower distress specifically during ER with acceptance.

**Table 1:** Relationship between self-reported distress levels during ER and resting and task HRV (correlation coefficients, uncorrected and FWE-corrected *p*-values).

Emotion Regulation Strategy (self-reported distress ratings)	Resting HRV (ms^2^)	Task HRV (ms^2^)	Correlation of resting and task HRV during respective task condition
ER with reappraisal			
*Spearman’s ρ (p-value; FWE p-value)*	−0.23 (0.051; 0.051)	−0.14 (0.16; 0.32)	0.88 (< 0.001; 0.002)
ER with acceptance			
*Spearman’s ρ (p-value; FWE p-value)*	−0.27 (0.026; 0.052)	−0.13 (0.17; 0.17)	0.86 (< 0.001; 002)
Emotion permitting			
*Spearman’s ρ (p-value)*	−0.15 (0.14)	−0.10 (0.23)	0.82 (< 0.001)

*First column*: the correlational analyses revealed a negative association between distress levels during ER with acceptance and resting HRV, both associations (resting HRV with reappraisal and acceptance) remained non-significant at threshold significancy after FWE corrections. *Second column*: for task HRV, correlational analyses revealed a negative nonsignificant association between distress levels during ER with reappraisal and ER with acceptance and task HRV. Thus, task HRV levels were not associated with distress levels during ER. *Third column*: resting and task HRV showed a strong positive association, suggesting that subjects with higher resting HRV also show higher task HRV in all experimental conditions.

The correlational analyses on the relationship between emotion regulation and task HRV showed a nonsignificant association between distress ratings (self-reported) and task HRV for both ER strategies and emotion permit (Table 1, second column).

The correlational analyses on the relationship between resting HRV and task HRV indicated a significant association between resting HRV and task HRV during both ER strategies and emotion permitting (Table 1, third column). This suggests that subjects with higher resting HRV have significantly higher task HRV while regulating and permitting emotions.

### fMRI

#### Brain correlates of ER with reappraisal and ER with acceptance

Comparisons of ER with reappraisal to a baseline condition (ER_permit) resulted in a differential brain activation in a cluster in the left lingual gyrus (See suppl. Material, Table S3). Contrasting ER with acceptance to ER_permit resulted in a differential brain activation in a cluster in the right inferior temporal lobule, among others (See suppl. Material, Table S4).

#### Specific brain correlates of ER with reappraisal versus ER with acceptance

**Figure 2:**
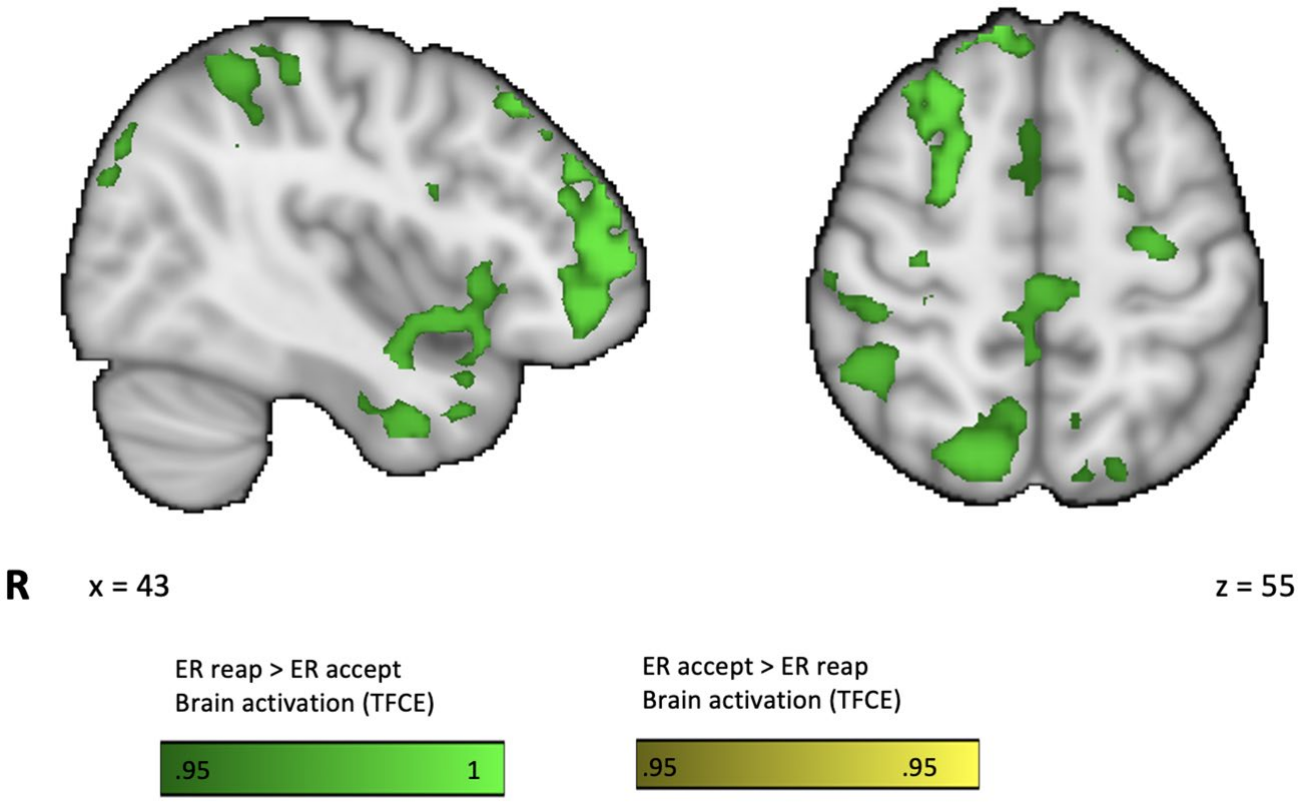
Brain correlates of emotion regulation with reappraisal vs acceptance (ER_reap vs ER_accept contrast) brain activation for fMRI BOLD signal depicted as thresholded and corrected *p* values < 0.05 (TFCE ranges 0.95–1), revealed differential brain activation in regions associated with emotion regulation and cognitive control.

Analyses of the specific brain correlates of ER_reap > ER_accept revealed increased brain activations in a cluster in the R frontal pole, extending into DLPFC, superior parietal lobule (SPL), L anterior insula and cingulate cortices, among others (See Fig. 2; Table 2). There were no significant activations for the contrast ER_accept > ER_reap.

**Table 2:** Functional brain activation for the contrast ER_reap > ER_accept.

Brain region	H	CS	MNI Coordinates			PE max	1 − p Max
			X	Y	Z		
FP, IFG, DLPFC, SPL	R	12,062	30	62	24	5.79	0.997
Parahippocampal gyrus	L	3513	−28	−40	−12	5.66	0.998
FP	L	939	−36	54	22	4.74	0.99
Caudate Nucleus	L	512	−7	16	8	5.5	0.999
Anterior Insula	L	291	−38	14	−12	4.37	0.965
Anterior Cingulate	L	286	−2	33	5	3.74	0.986
Precuneus	R	195	22	−54	22	4.05	0.986
SFG, SMA	R	159	2	14	56	3.42	0.959
MFG, FP	L	141	−46	52	4	3.27	0.956
Mid Anterior Cingulate	R	62	4	14	22	2.97	0.988
Posterior Cingulate	R	21	6	−18	26	2.79	0.984

Results are derived from a factorial GLM contrasting ER_reap vs ER_accept. All results are thresholded and corrected for multiple comparisons with adjusted *p* value < 0.05 (equivalent to 0.95–1 using TFCE; 1 − p Max). * Only cluster overpassing 20 k are reported. H, hemisphere; CS, cluster size in the number of activated voxels; L, left; R, right; FP: frontal pole; IFG: inferior frontal gyrus; DLPFC: dorso-lateral prefrontal cortex; SPL: superior parietal lobule; SFG: superior frontal gyrus; SMA: supplementary motor area; PE: parameter estimate; PE max: maximum value of the PE for the cluster.

### Brain and task HRV associations

#### Associations between brain activation during ER with reappraisal and task HRV

**Figure 3:**
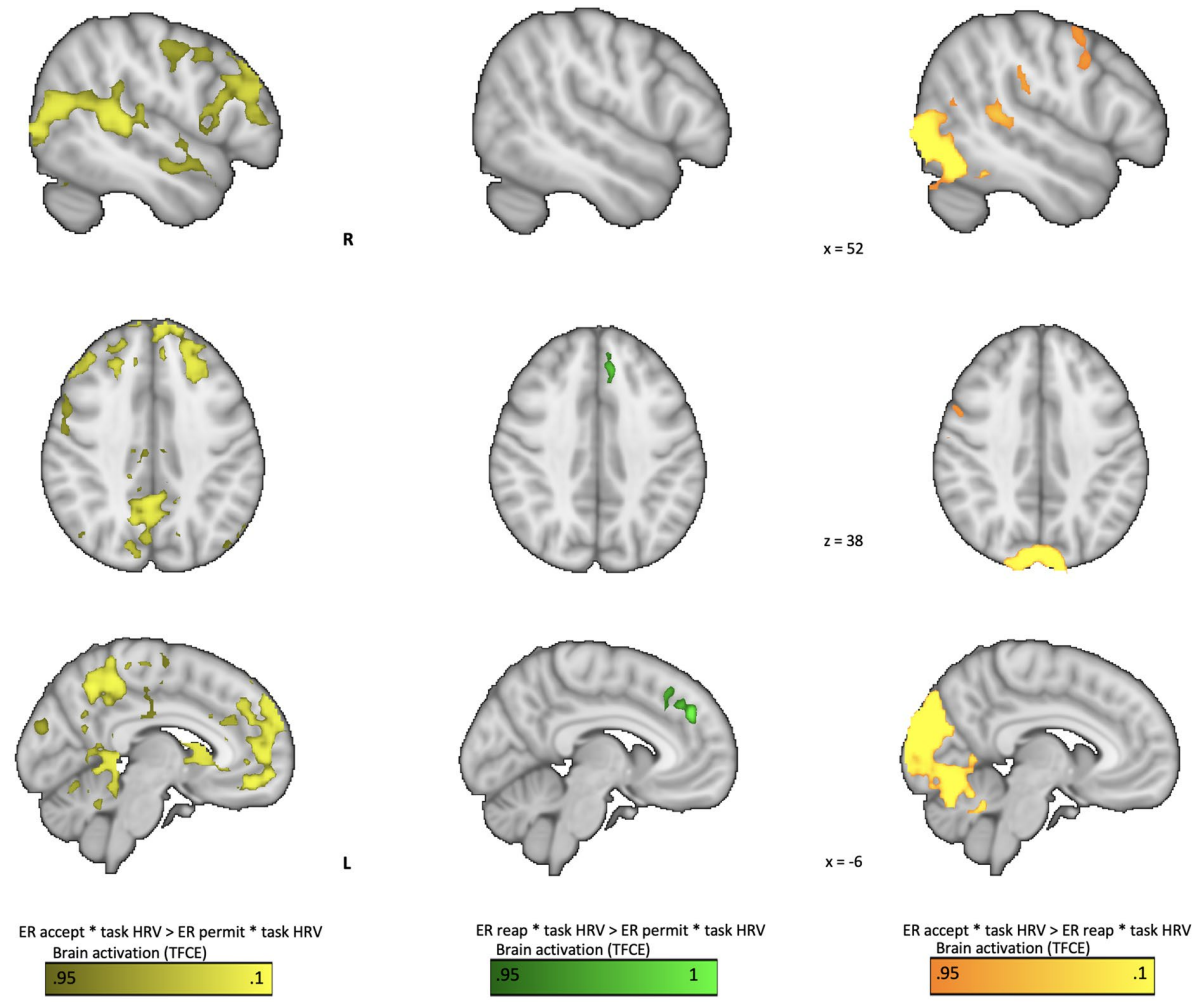
Covariation of task HRV and brain activation during emotion regulation: *in light-yellow* the contrast acceptance vs permit, *in green* the contrast reappraisal vs permit, *in warm-yellow* the contrast acceptance vs reappraisal. Parametric regression of task HRV and whole-brain activation for fMRI BOLD signal depicted as thresholded and corrected *p* values < 0.05 (TFCE ranges 0.95–1). The contrast reappraisal vs permit revealed higher covariation of task HRV and brain activation in the DMPFC, while for acceptance vs permit there was higher covariation in several regions of the mid-line brain including DMPFC, vACC, basal ganglia, these regions have been associated with ER and HRV modulation. For acceptance vs reappraisal, there was higher covariation in several regions linked to self and sensory-motor processing.

A comparison of the contrasts ER_reap*task HRV and ER_permit*task HRV showed that higher activation in the dorsal part of the medial PFC was associated with higher HRV (See Fig. 3; Table 3).

**Table 3:** Covariation of task HRV and brain activation during emotion regulation for the contrast ER_reap > ER_permit.

Brain region	H	CS	MNI Coordinates			PE max	1 − p Max
			X	Y	Z		
Dorso medial PFC	L	235	−4	44	30	5.16	0.969

Results are derived from a factorial GLM contrasting ER_reap vs ER_permit and task HRV as parametric regressor. All results are thresholded and corrected for multiple comparisons with adjusted *p* value < 0.05 (equivalent to 0.95–1 using TFCE; 1 − p Max). * Only cluster overpassing 20 k are reported in this table. H, hemisphere; CS, cluster size in the number of activated voxels; L, left; R, right; PFC: prefrontal cortex. PE: parameter estimate; PE max: maximum value of the PE for the cluster.

#### Associations between brain activation during ER with acceptance and task HRV

Comparison of the contrasts ER_accept*task HRV and ER_permit*task HRV showed that higher activation in extensive regions of mid line brain regions including the VMPFC, DMPFC, ACC, SMA, and precuneus as well as bilateral TPJ was associated with higher HRV (See Fig. 3; Table 4).

**Table 4:** Covariation of task HRV and brain activation during emotion regulation for the contrast ER_accept > ER_permit.

Brain region	H	CS	MNI Coordinates			PE max	1 − p Max
			X	Y	Z		
DMPFC, VMPFC, ACC, SMA, DLPFC, Precuneus, others	R	21,001	6	−50	50	6.34	0.996
TPJ, SMG	L	749	−60	−42	14	4.36	0.977
Postcentral gyrus	R	303	54	−10	44	3.71	0.969
LOC	L	251	−38	−66	28	4.18	0.964
Occipital pole	L	132	−4	−90	20	4.92	0.97
Central Opercular C	R	33	−56	−2	6	3.63	0.954
OFC, IFG	R	28	26	34	−16	3.04	0.971

Results are derived from a factorial GLM contrasting ER_accept vs ER_permit and task HRV as parametric regressor. All results are thresholded and corrected for multiple comparisons with adjusted *p* value < 0.05 (equivalent to 0.95–1 using TFCE; 1 − p Max). * Only cluster overpassing 20 k are reported here. H, hemisphere; CS, cluster size in the number of activated voxels; L, left; R, right; DMPFC: dorso-medial prefrontal cortex; VMPFC: ventro-medial prefrontal cortex; ACC: anterior cingulate cortex; SMA: supplementary motor area; DLPFC: dorso-lateral prefrontal cortex; TPJ: temporo-parietal junction; SMG: supramarginal gyrus; LOC: lateral occipital cortex; OFC: orbito frontal cortex; IFG: inferior frontal gyrus; PE: parameter estimate; PE max: maximum value of the PE for the cluster.

#### Associations of brain activation and task HRV in reappraisal vs acceptance

To investigate differential neuro-physiological interactions during ER_reap and ER_accept, we compared covariation maps from ER_reap*task_HRV > ER_accept*task_HRV, which resulted in no significant brain activation. In contrast, ER_accept*task_HRV > ER_reap*task_HRV resulted in a differential brain activation in a cluster in the right TPJ, IPL, bilateral fusiform gyrus, and pre and postcentral gyrus (see Fig. 3; Table 5), suggesting a higher covariation of task HRV and brain activation during acceptance compared to reappraisal.

**Table 5:** Covariation of task HRV and brain activation during emotion regulation for the contrast ER_accept > ER_reap.

Brain region	H	CS	MNI Coordinates			PE max	1 − p Max
			X	Y	Z		
TPJ, IPL, LOC, TO-FG, OC,	R	20,783	6	−66	−28	13.7	1
Precentral gyrus	R	110	54	2	52	4.04	0.961
Postcentral gyrus	L	50	−42	−22	42	4.6	0.961
Postcentral gyrus	R	42	62	−6	44	3.88	0.956
Precentral gyrus	R	31	−56	−2	6	3.96	0.956

Results are derived from a factorial GLM contrasting ER_accept vs ER_permit and task HRV as parametric regressor. All results are thresholded and corrected for multiple comparisons with adjusted *p* value < 0.05. * Here, only cluster overpassing 20 k are reported. H, hemisphere; CS, cluster size in the number of activated voxels; L, left; R, right; TPJ: temporo-parietal junction; IPL: inferior parietal lobule; LOC: lateral occipital cortex; TO-FG: temporo-occipital fusiform gyrus; OC: occipital cortex; PE: parameter estimate; PE max: maximum value of the PE for the cluster.

### Brain and resting HRV associations

#### Associations between brain activation during ER with reappraisal and resting HRV (high vs low resting HRV group)

The group comparison (high vs low resting HRV) of the contrast ER_reap > ER_permit showed that during ER with reappraisal, subjects with higher resting HRV did not show higher brain activation compared to those with low resting HRV.

#### Associations between brain activation during ER with acceptance and resting HRV (high vs low resting HRV group)

The group comparison (high vs low resting HRV) of the contrast ER_accept > ER_permit showed that during ER with acceptance subjects with higher resting HRV did not show higher brain activation compared to those with low resting HRV.

#### Effects of ER strategies and resting HRV (high vs low resting HRV group) on pre-SMA activation (ROI analysis)

**Figure 4:**
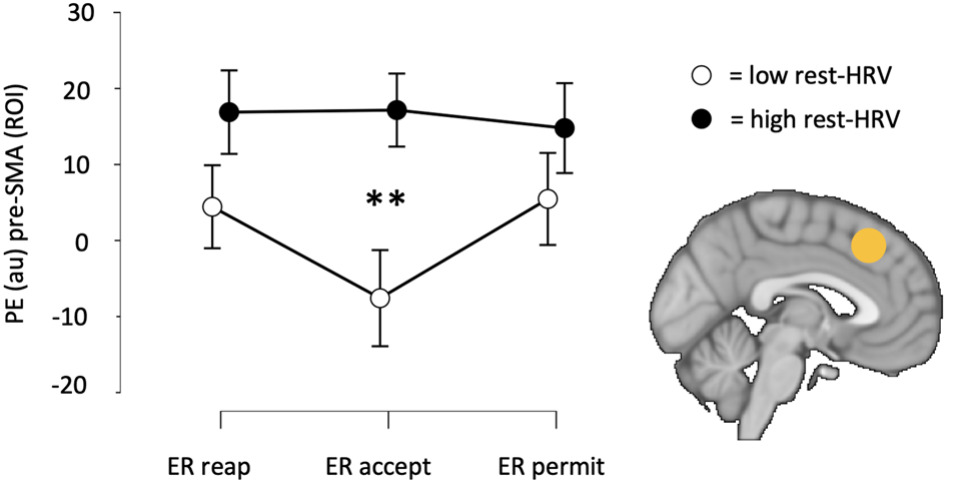
Mean pre-SMA activation during emotion regulation using reappraisal (ER_reap), acceptance (ER_accept) or permit (ER_permit), for low and high resting HRV groups. Error bars correspond to 95% confidence intervals. Pairwise comparisons with FWE correction (**p < 0.01) confirmed a main effect of Group: group with high resting HRV showed higher pre-SMA activation than the low HRV group during the ER task. A significant Strategy × Group interaction revealed that specially during ER_accept pre-SMA activation was higher in the high resting HRV group. The brain image depicts the location for the pre-SMA PE extraction, according to pre-defined MNI coordinates − 2, 14, 58. PE: parameter estimate; AU: arbitrary units; ROI: regions of interesting.

The repeated measures ANOVA yielded no significant main effect of Strategy (*F*_1,108_ = 2.71, *p* = 0.071, *η*^2^_*p*_ = 0.048). A main effect of Group (*F*_1,54_ = 6.62, *p* = 0.013, *η*^2^_*p*_ = 0.109) showed that pre-SMA activation was higher in the group with high resting HRV compared to low resting HRV (Fig. 4). A significant interaction effect of Strategy × Group (*F*_1,108_ = 4.27, *p* = 0.016, *η*^2^_*p*_ = 0.073) showed that specifically during ER acceptance pre-SMA activation was higher in the high resting compared to the low resting HRV group (corrected *p* = 0.007). These findings suggest that the high resting HRV group has higher pre-SMA activation during ER compared to the low resting HRV group, especially during regulating emotions with acceptance.

## Discussion

The present study used an emotion regulation fMRI paradigm to investigate mechanisms linking resting and task HRV to ER in behavioral and brain processes. According to our hypothesis, we found that high resting HRV was associated with increased task HRV during emotion regulation during both reappraisal and acceptance, as well as during permitting of emotions. Furthermore, we found that numerically, individuals exhibiting high levels of resting HRV experienced lower personal distress when regulating emotions with acceptance, although this result did not survive correction for multiple comparisons. Furthermore, task HRV and personal distress associations could not be found. Interestingly, fMRI parametrical modulation analyses revealed that higher task HRV was associated with increased dorso-medial PFC activation (for reappraisal vs permit), and with extensive middle line brain regions, including DMPFC and vACC and precuneus (for acceptance vs permit). Concomitantly, subjects with higher resting HRV compared to low resting HRV showed higher activation in pre-SMA during ER using an ROI approach. This study demonstrates that both resting and task HRV are associated with ER brain activation, albeit somewhat differentially, pointing to specific brain mechanisms for resting and task HRV.

### Linking emotion regulation and resting and task HRV

Resting HRV was associated with lower distress during ER across ER strategies only in a numerical fashion and results did not survive correction for multiple comparisons; we therefore failed to replicate findings that subjects with higher resting HRV have better capacities for regulating negative emotions^22–24^. However, it is important to note that the association of resting HRV and emotion regulation with acceptance was significant before correction for multiple comparisons, in line with studies investigating only one emotion regulation strategy^29^. This finding suggests that subjects with high resting HRV might benefit more from using acceptance and less so from reappraisal as ER strategy. This supports the notion of acceptance being an adaptive ER strategy^6,43^, and to the best of our knowledge, this is the first time this ER strategy is linked to high levels of resting HRV.

In this study we could not show that task HRV was linked to a higher capacity for regulating negative emotions, as previous literature suggests^24^. Also, task HRV did not differ across the ER strategies and the baseline condition as previous studies have suggested^24^. Why task HRV was not higher during ER (compared to baseline), and why it was not associated to lower distress during ER can only be answered speculatively. During ER different cognitive processes might have opposite effects on task HRV, as a cognitively demanding task some processes can be linked to a decrease in task HRV (e.g. for maths tasks cognitive load increases the sympathetic tone, thus decreases task HRV), while other processes could be linked to increases in task HRV (i.e. direct modulation of current emotional task). Thus, it might be that task HRV and personal distress (ER) are negatively associated, although with a small effect size. Thus, our study might have had insufficient power to detect it, that is, its relationship might be weaker compared to resting HRV and ER associations.

Resting and task HRV were positively and strongly correlated, indicating that subjects with higher resting HRV have higher task HRV during actively regulating negative emotions. To the best of our knowledge, this is the first time both metrics are investigated together, proving initial convergent validity for task HRV as a HRV estimate. Notably, in the current study, task HRV was recorded during the ER task, averaging trials of 6 s. This brief acquisition window may not reliably capture the high-frequency spectrum of the HRV signal^44^. Nonetheless, the robust correlation between resting and task HRV estimates supports our current approach to measuring task HRV. Future research should endeavor to further validate short phasic HRV estimates.

### Neuro-physiological mechanisms linking emotion regulation and resting and task HRV

Task HRV and resting HRV (only at an ROI level), were both positively associated with ER brain activation. The results highlighted a role for DMPFC and midline regions in both HRV and ER, which is coherent with previous studies and models, e.g. neuro-visceral integration (Thayer et al., 2012; Thayer & Lane, 2000).

Since previous studies only linked resting HRV and ER brain processes, our study is the first to investigate the relation of task HRV and ER brain processes. In line with our hypotheses, both acceptance and reappraisal, compared to baseline, showed higher covariation of task HRV and midline brain regions, suggesting a key role for these regions in modulating ongoing HRV during ER processing. In the context of ER brain networks, this finding hints towards a more regulatory role of DMPFC and pre-SMA regions, as these might be implicated in generating regulated emotional tasks^25^.

Contrasting reappraisal with acceptance as single contrasts resulted in higher activation in areas such as the DLPFC and IFG, while the reverse contrast of acceptance vs reappraisal did not yield activation differences, replicating a previous study comparing both strategies^7^. This result is coherent with the notion of acceptance being a less resource-consuming strategy as compared to reappraisal, in which subjects need to deliberately change the cognitive or semantic frame of stressful stimuli^43^. Even more, comparing acceptance and reappraisal using task HRV-brain covariation maps showed a differential engagement of somato-sensory and motor regions (right TPJ, IPL, pre-central cortex, etc.) in ongoing modulation of HRV specific for acceptance, suggesting a role in ER. This is coherent with embodied accounts of ER, which posit a central role for somato-sensory and interoceptive regions in ER processing^45^, and with the notion that acceptance entails a more direct engagement with current emotional and bodily experience^5,6^. Interestingly, the idea of the patient learning to accept negative physiological arousal without directly needing to act upon it, forms a central aspect of the psychotherapy method of ACT^46^, while their reframing in terms of learning new stimulus–response associations triggering these negative emotions forms the central learning mechanism within CBT^47^. Therefore, our results show a different implementation and interaction of brain and physiological processes, which is coherent with psychological and psychotherapeutical accounts and grounding principles of both as different ER strategies for reappraisal^5^, for acceptance see^46^.

The question emerges why resting HRV was not associated with higher brain activation in ER regions. Here we failed to replicate previous studies, despite taking different methodological approaches, i.e., whole-brain analyses and group splitting based on a bi-modal distribution. Only when using ROI analysis, with a predefined ER midline region (SMA), subjects with higher resting HRV showed higher brain activation in SMA, as in previous studies^29^. Contrary to Steinfurth et al., 2018, we did not find that higher resting HRV was linked to DMPFC, but instead to pre-SMA. This could be due to several methodological differences such as statistical power (sample size), reporting of uncorrected alpha levels (*p*-values) in previous studies, and predefined selection of ROIs. In the present study, using whole-brain and corrected analyses, we could show the involvement of DMPFC in ER and task HRV, thus suggesting a key role for this region in linking both processes. Furthermore, our results suggest a role for pre-SMA in ER and resting HRV, suggesting that subjects with higher resting HRV might have higher activation of this region, known to be involved in agency and self-directed behavior^48–50^.

Putting together our findings, they suggest that during ER subjects with higher resting HRV have higher task HRV and are more descriptively successful in regulating their emotions, which in turn seems to be mediated by mid-line frontal cortex regions, like the dorso-medial PFC and pre-SMA. Thus, our findings demonstrate a plausible brain mechanism linking HRV and ER processes. Finally, despite investigating the linkage of rest and task HRV with self-reported distress and rest and task HRV with brain activation, in this publication, brain activity and distress levels associations were not explored.

### Implications for clinical psychology and medicine

Reappraisal and acceptance are both recognized as adaptive ER strategies^4^, and are part of some of the most widely used psychotherapy approaches, i.e., CBT and ACT. Considering the literature and our current findings, it might be that both strategies are preferentially applied in different situations, for example, reappraisal might be the strategy of choice when dealing with externally induced stressful events like threat situations, i.e., in situationally induced panic attacks, while acceptance might be better suited to deal with more internally challenging situations like having to deal with a life change, i.e., the death of a close relative. Our current study advances the understanding of underlying neuro-physiological mechanisms of ER, and in this way of psychological interventions based on these strategies^8^.

Several mental disorders, like mood disorders, that are characterized by emotion regulation deficits show reduced HRV levels^13–15^. Our findings could help to further the development of neurobiologically informed treatments, like transcranial magnetic stimulation (TMS), for which targeting midline ER regions (like DMPFC) might induce personal distress reduction together with task HRV changes. A similar approach has been already tested, proposing task HRV as a marker of ongoing success of ER through TMS therapy for mood disorders^51^.

Recently, anxiety disorders have been described as having a heightened autonomic sensitivity, linked to higher sympathetic response together with a blunted medial frontal brain activity^52^. Our results provide a coherent frame for understanding the interaction between cardiac modulation, midline brain activation and emotional distress, thus highlighting the role of emotion regulation for reducing distress, while modulating midline brain regions and the parasympathetic tone, i.e., task HRV.

### Limitations

There are several strengths and limitations of our study. The study of HRV and emotion regulation and their brain underpinnings in a concomitant fashion has only relatively recently started and to the best of our knowledge our study to date includes the largest group of subjects, thus increasing its explanatory power. Importantly, among the 62 subjects, 52 were female, which might have influenced the results and reduces their generalizability given also that gender differences have been reported for emotion regulation^53^. Regarding the ERT design, despite the inclusion of the aversive sound and red pumping dots aimed at enhancing realism and ecological validity, which were correctly counterbalanced, they may have induced overall higher arousal and distress, potentially influencing brain activation and HRV task correlates. These may affect the comparability of our findings with respect to previous works in the emotion regulation field. Even though the vocalization of strategies was performed across all conditions and motion artifacts were correctly controlled, this factor may have still influenced brain and HRV parameters. Future studies could investigate other strategies for ER, such as different forms of cognitive reappraisal (i.e. positive reframing), but also to take into account different learning rates of ER strategies (i.e. it might be that using acceptance as a strategy requires longer periods of training). Further studies should take into account individual differences in emotion regulation and emotion processing to better characterize factors or traits that moderate the relationship between emotion regulation and HRV. Other contextual factors including age, ethnicity could have potentially influenced our findings. Furthermore, considering the known effects of menstrual cycles, circadian rhythms, hunger, and satiety on emotion processing and HRV, it is possible that these physiological factors influenced both emotion and HRV processing. This is especially relevant for the effect of menstrual cycles (which were not acquired), given the large representation of female participants in our sample. Finally, new studies could take advantage of the ERT exploring how HRV and emotion regulation varies across diverse clinical populations of known emotional and social dysfunction (e.g. borderline personality disorder, autism and schizophrenia spectrum conditions).

## Conclusions

The current study sheds light on the psychological and brain mechanisms of resting and task HRV. Our findings expand the current literature by showing a positive correlation of resting and task HRV, and that the mid-line frontal cortex, dorso-medial PFC are pre-SMA are specially linked to task HRV and resting HRV during ER, thus providing evidence for a brain mechanism linking resting and task HRV with ER. Furthermore, and in line with previous research we show that high levels of resting HRV are descriptively associated with low levels of personal distress during ER with acceptance.

### Supplementary Information

Supplementary Information.

## Data Availability

All data is available upon request, please direct your email to the corresponding author: simon.guendelman@hu-berlin.de.
