# Supplementary material for: Brain mechanisms underlying the modulation of heart rate variability when accepting and reappraising emotions
Source: Sci Rep. 2024 Aug 13;14:18756. doi: 10.1038/s41598-024-68352-4 (PMC11322180; doi:10.1038/s41598-024-68352-4)
Supplement: Supplementary file 1 — Supplementary Information. [file 41598_2024_68352_MOESM1_ESM.docx]

**Supplemental Material:**

**Table S1.** Block type, including relative order (number) and corresponding strategy when aversive sound was delivered.

Version 1:

5 aversive sounds

| Block Number | ER_self or ER_other | Block Strategy |
| --- | --- | --- |
| 1 | ER_self | MFN |
| 5 | ER_other | REAP |
| 10 | ER_other | MFN |
| 16 | ER_other | REAP |
| 19 | ER_self | REAP |

Version 2:

4 aversive sounds

| Block Number | ER_self or ER_other | Block Strategy |
| --- | --- | --- |
| 2 | ER_self | REAP |
| 9 | ER_self | MFN |
| 12 | ER_other | REAP |
| 20 | ER_self | MFN |

Version 3:

3 aversive sounds

| Block Number | ER_self or ER_other | Block Strategy |
| --- | --- | --- |
| 6 | ER_other | MFN |
| 7 | ER_self | REAP |
| 18 | ER_other | MFN |

**Table S2.** Selected pictures from the International affective picture system (IAPS):

Considering normative ratings (arousal and valence) of IAPS pictures, and us suggested by (Lang et al., 2008), we determined neutral pictures as having valence ratings between 4.5 - 5.5 and arousal ratings lower than 3.6. Negative low arousal, valence lower than 4.1 and arousal between 5.6 - 6.4; negative high arousal, valence lower than 4.1 and arousal major than 6.4.

Lang, P.J., Bradley, M.M., & Cuthbert, B.N. (2008). International affective picture system (IAPS): Affective ratings of pictures and instruction manual. Technical Report A-8. University of Florida, Gainesville, FL.

| Neutral | Negative low arousal | Negative high arousal |
| --- | --- | --- |
| 2190 | 1019 | 1050 |
| 2200 | 1040 | 1052 |
| 2210 | 1051 | 1120 |
| 2214 | 1070 | 1300 |
| 2215 | 1090 | 1525 |
| 2357 | 1110 | 1931 |
| 2381 | 1113 | 2730 |
| 2383 | 1200 | 3000 |
| 2393 | 1201 | 3000 |
| 2480 | 1205 | 3010 |
| 2493 | 1220 | 3010 |
| 2495 | 1301 | 3030 |
| 2499 | 1930 | 3053 |
| 2514 | 1932 | 3060 |
| 2516 | 2661 | 3068 |
| 2570 | 2683 | 3069 |
| 2720 | 2688 | 3071 |
| 2840 | 2691 | 3080 |
| 2850 | 2981 | 3102 |
| 2870 | 3015 | 3110 |
| 2880 | 3022 | 3120 |
| 2890 | 3051 | 3130 |
| 5500 | 3062 | 3150 |
| 5510 | 3063 | 3170 |
| 5520 | 3064 | 3266 |
| 5530 | 3100 | 3400 |
| 5533 | 3101 | 3500 |
| 5534 | 3140 | 3530 |
| 5731 | 3168 | 5971 |
| 5740 | 3180 | 6230 |
| 6150 | 3220 | 6250 |
| 7000 | 3250 | 6260 |
| 7002 | 3261 | 6300 |
| 7004 | 3350 | 6313 |
| 7006 | 3550 | 6350 |
| 7009 | 5972 | 6510 |
| 7010 | 6020 | 6540 |
| 7020 | 6190 | 6550 |
| 7025 | 6200 | 6560 |
| 7030 | 6200 | 9250 |
| 7031 | 6210 | 9252 |
| 7034 | 6211 | 9410 |
| 7035 | 6212 | 9810 |
| 7036 | 6213 | 9921 |
| 7038 | 6243 | 6250.1 |
| 7040 | 6244 | 9635.1 |
| 7041 | 6312 | 2811 |

Actual standardized values for valence were: for negative high arousal pictures (M= 3.13, SD=0.14), negative low arousal pictures (M= 2.94, SD=0.85), neutral pictures (M= 5.01, SD=0.24). Standardized values for arousal were: for negative high arousal pictures (M= 6.87, SD=0.24), negative low arousal pictures (M= 5.98, SD=0.28), neutral pictures (M= 2.90, SD=0.41).

One-way ANOVA were used to compare the pictures. Regarding valence, the one-way ANOVA resulted in a statistically significant main effect of valence (F2-77 = 1036; p

< .001). Post hoc tests showed a significant difference between pictures was found when comparing neutral with negative high arousal (T74= - 45.4, p <.001), and with negative low arousal (T74= - 15.9, p <.001), while negative high and low arousal pictures were non different (T74= 1.54, p =0.28). As intended, this indicates lower

(negative) valence for pictures with negative high and low arousal compared with neutral pictures.

Regarding arousal, the one-way ANOVA resulted in a statistically significant main effect of arousal (F2-88 = 1598; p < .001). Post hoc tests showed a significant difference between pictures was found when comparing neutral with negative high arousal (T74= 56.7, p <.001), and with negative low arousal (T74= 42.5, p <.001), while negative high and low arousal pictures were also significantly different (T74= 16.4, p <.001). As intended, this indicates higher arousal when comparing negative low arousal with neutral pictures, and also when comparing negative high with negative low arousal pictures.


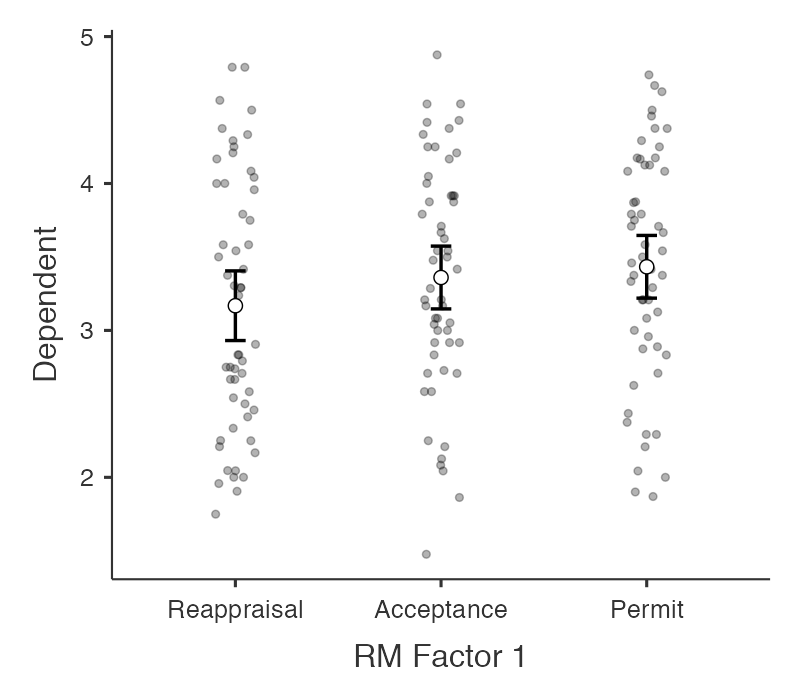


**Figure S1.** Self reported distress levels across the three emotion regulation strategies, reappraisal, acceptance and permit.


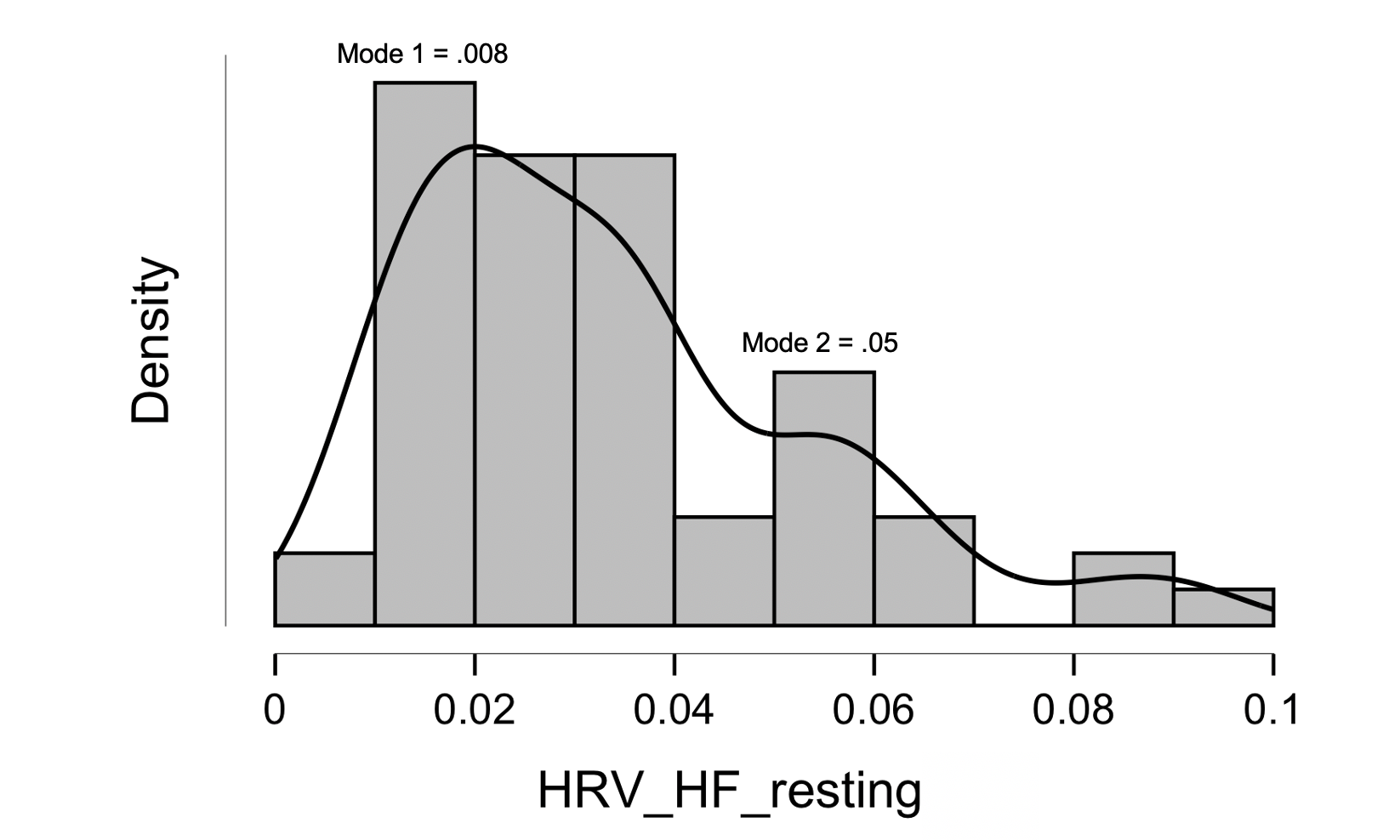


**Figure S2.** Descriptive plot of density of nonnormal distribution of trait HRV values. Two modes were detected.

**Table S3**. Functional brain activation during emotion regulation for the contrast ER_reap > ER_permit.

| **Brain region** |  |  | **MNI Coordinates** | | |  |  |
| --- | --- | --- | --- | --- | --- | --- | --- |
|  | H | CS | X | Y | Z | PE max | 1 – p Max |
| Lingual gyrus | L | 53 | -26 | -46 | -6 | 5.25 | 0.961 |
|  |  |  |  |  |  |  |  |

Note: results are derived from a factorial GLM contrasting ER_reap vs ER_permit. All results are thresholded and corrected for multiple comparisons with adjusted *p value* <0.05 (equivalent to .95-1 using TFCE; 1 – p Max). * Only cluster overpassing 20 *k* are reported. Abbreviations: H, hemisphere; CS, cluster size in the number of activated voxels; L, left; R, right; PFC: prefrontal cortex. PE: parameter estimate; PE max: maximum value of the PE for the cluster.

**Table S4**. Functional brain activation during emotion regulation for the contrast ER_accept > ER_permit.

| **Brain region** |  |  | **MNI Coordinates** | | |  |  |
| --- | --- | --- | --- | --- | --- | --- | --- |
|  | H | CS | X | Y | Z | PE max | 1 – p Max |
| Lateral occipital cortex | R | 69 | 52 | -68 | 8 | 6.45 | 0.974 |
|  |  |  |  |  |  |  |  |

Note: results are derived from a factorial GLM contrasting ER_accept vs ER_permit. All results are thresholded and corrected for multiple comparisons with adjusted *p value* <0.05 (equivalent to .95-1 using TFCE; 1 – p Max). * Only cluster overpassing 20 *k* are reported. Abbreviations: H, hemisphere; CS, cluster size in the number of activated voxels; L, left; R, right; PFC: prefrontal cortex. PE: parameter estimate; PE max: maximum value of the PE for the cluster.
